# Inflammatory and Haematological Markers in the Maternal, Umbilical Cord and Infant Circulation in Histological Chorioamnionitis

**DOI:** 10.1371/journal.pone.0051836

**Published:** 2012-12-13

**Authors:** Rebecca A. Howman, Adrian K. Charles, Angela Jacques, Dorota A. Doherty, Karen Simmer, Tobias Strunk, Peter C. Richmond, Catherine H. Cole, David P. Burgner

**Affiliations:** 1 School of Paediatrics and Child Health, University of Western Australia, Perth, Western Australia, Australia; 2 Women and Infants Research Foundation, Perth, Western Australia, Australia; 3 School of Women’s and Infants’ Health, University of Western Australia, Perth, Western Australia, Australia; 4 Murdoch Childrens Research Institute, Royal Children’s Hospital, Parkville, Victoria, Australia; 5 Department of Paediatrics, University of Melbourne, Melbourne, Victoria, Australia; The Ohio State Unversity, United States of America

## Abstract

**Background:**

The relationship between histological chorioamnionitis and haematological and biochemical markers in mothers and infants at delivery, and in infants postnatally, is incompletely characterised. These markers are widely used in the diagnosis of maternal and neonatal infection. Our objective was to investigate the effects of histological chorioamnionitis (HCA) on haematological and biochemical inflammatory markers in mothers and infants at delivery, and in infants post-delivery.

**Methods:**

Two hundred and forty seven mothers, delivering 325 infants, were recruited at the only tertiary perinatal centre in Western Australia. Placentae were assessed for evidence of HCA using a semi-quantitative scoring system. Maternal high sensitivity C-reactive protein (hsCRP), procalcitonin, and umbilical cord hsCRP, procalcitonin, white cell count and absolute neutrophil count were measured at delivery. In infants where sepsis was clinically suspected, postnatal CRP, white cell count and absolute neutrophil count were measured up to 48 hours of age. The effect of HCA on maternal, cord and neonatal markers was evaluated by multivariable regression analysis.

**Results:**

The median gestational age was 34 weeks and HCA was present in 26 of 247 (10.5%) placentae. Mothers whose pregnancies were complicated by HCA had higher hsCRP (median 26 (range 2–107) versus 5.6 (0–108) mg/L; *P*<0.001). Histological chorioamnionitis was associated with higher umbilical cord hsCRP (75^th^ percentile 2.91 mg/L (range 0–63.9) versus 75^th^ percentile 0 mg/L (0–45.6); *P<*0.001) and procalcitonin (median 0.293 (range 0.05–27.37) versus median 0.064 (range 0.01–5.24) ug/L; *P<*0.001), with a sustained increase in neonatal absolute neutrophil count (median 4.5 (0.1–26.4)×10^9^/L versus 3.0 (0.1–17.8)×10^9^/L), and CRP up to 48 hours post-partum (median 10 versus 6.5 mg/L) (*P<*0.05 for each).

**Conclusion:**

Histological chorioamnionitis is associated with modest systemic inflammation in maternal and cord blood. These systemic changes may increase postnatally, potentially undermining their utility in the diagnosis of early-onset neonatal infection.

## Background

Intrauterine infection and inflammation (IUI) is an important determinant of spontaneous preterm birth [1], [2]. Infants whose pregnancies were complicated by IUI have increased risk of adverse outcomes including perinatal death, early onset neonatal sepsis, septic shock, pneumonia, intraventricular hemorrhage, cerebral white matter damage, and long-term disability such as cerebral palsy [1], [3], [4]. The fetal response to IUI is characterised by increased levels of proinflammatory cytokines in the amniotic fluid and cord blood [4], [5], and by inflammation of the chorionic plate, placental vessels and/or umbilical cord (fetal vasculitis) [6], [7]. However the relationships between perinatal inflammation and inflammatory markers in umbilical cord blood, infant and maternal circulation are incompletely understood.

Intrauterine infection and inflammation is most accurately diagnosed by histological examination of the placenta. Histological chorioamnionitis (HCA) is often asymptomatic and clinical signs, such as fever, uterine tenderness, maternal or fetal tachycardia and malodorous amniotic fluid, lack both sensitivity and specificity; over one third of women with these signs do not have histological evidence of placental inflammation [5], as other causes may produce similar clinical features [6]. Moreover intrauterine infection occurs without microbiologically-proven amniotic fluid infection in over half of cases [7], due to due to fastidious organisms, sampling limitations and prior exposure to antibiotics. Molecular techniques for fastidious microorganisms may increase the diagnostic yield, but are not widely available and their sensitivity and specificity are unknown. The poor predictive value of clinical signs and amniotic fluid culture for identifying HCA has increased interest in readily available inflammatory and haematological markers.

C-reactive protein (CRP), procalcitonin (PCT) and absolute neutrophil count (ANC) are biomarkers that may be used as adjunctive tests in the diagnosis of inflammation, sepsis and infection. CRP is an acute phase reactant produced by the liver in response to pro-inflammatory cytokines, such as interleukin (IL)-6. Plasma levels increase 12–24 hours after the onset of inflammation, when diagnostic clinical signs may be non-specific, and remains elevated until after stimulus resolves [8]. Along with ANC, CRP is a well-described marker for bacterial infection in neonates, children and adults. Elevation of serum CRP in the neonate is due to endogenous hepatic synthesis, since very low quantities of CRP cross the placenta [9]. Procalcitonin (PCT) is a peptide prohormone of calcitonin physiologically produced by C-cells in the thyroid gland. In response to endotoxin and inflammatory cytokines, PCT it is also synthesised by non-thyroidal tissues, including monocytes, renal and pancreatic cells, adipose tissue, and hepatocytes [10].

Procalcitonin (PCT) may have advantages over CRP as a marker for inflammation or sepsis. PCT concentrations increase more rapidly than CRP (within 2–4 hours) in response to systemic inflammation or infection, remain elevated during the inflammatory process and rapidly return to baseline on resolution of the stimulus [10]. Thus PCT levels more accurately reflect the real-time extent of inflammation and may be more useful than CRP in the early diagnosis of infection and monitoring of disease. However, PCT appears to be of limited clinical utility in the neonatal setting as it may be elevated in non-infected infants with other complications such as asyphyxia, respiratory distress and intracranial haemorrhage [11].

Most data on inflammatory biomarkers in HCA relate to C-reactive protein (CRP) and routine haematological parameters; there is less experience with newer markers such as PCT, especially in the maternal circulation [12]. Few studies that have specifically investigated the relationship between cord and neonatal CRP in neonates exposed to HCA and the results are inconsistent [13], [14]. A single study of 8 subjects with HCA showed no difference in cord PCT concentrations in the presence of HCA [15]. Thus the associations between these markers and histologically confirmed chorioamnionitis are inconsistent and their clinical utility remains unclear [16].

The extent to which HCA may confound these diagnostic markers of neonatal sepsis is unknown, but HCA may affect inflammatory markers several days postpartum [15]. This is particularly pertinent in preterm infants, in whom pregnancies are most likely to be complicated by HCA, whose mothers are likely to have received intra-partum antibiotics (thereby reducing the yield of neonatal blood cultures), and who are at the highest risk of early life sepsis, which is difficult to diagnose clinically [11].

We hypothesised that HCA generates inflammatory changes that may be detected in the mother at the time of delivery and in the neonate up to 48 hours following delivery. Our aim was therefore to investigate the association of HCA and CRP, PCT and haematological parameters in the mother and baby at the time of delivery, and in the neonate up to 48 hours following delivery. We show that HCA has is associated with inflammatory responses in both the maternal circulation and cord blood and these may persist in the neonatal circulation postnatally. The presence of HCA should be considered when interpreting inflammatory indices in newborn preterm infants.

## Methods

### Study Population

This was a cross-sectional study of mother-infant pairs recruited at the only tertiary perinatal center (King Edward Memorial Hospital) in Western Australia (total population ∼2.2 million), between August 2003 and January 2006. We aimed to recruit women in preterm labour and those delivering at term in a ratio of approximately two to one. We enrolled women at deliveries attended by dedicated study personnel and consequently the study population was over-represented with multiple pregnancies and deliveries by caesarean section and under-represented with spontaneous vaginal deliveries of extreme preterm infants, whose deliveries frequently occur out-of-hours, when study personnel were unavailable. We excluded infants with major congenital abnormalities and mothers whose understanding of English did not allow fully informed consent. The study was approved by the King Edward Memorial Hospital for Women institutional ethics committee. All the subjects who were included in this study provided informed written consent.

### Definitions and Study Procedures

Gestational age was estimated from date of the last menstrual period, *in utero* ultrasound before 20 weeks, or in the absence of antenatal data, the Ballard score at delivery [17]. A section of umbilical cord, membranes and at least one section including the chorionic plate were analysed histologically. Histological chorioamnionitis was diagnosed by the presence of inflammatory cells (predominantly neutrophils) in the chorionic plate and/or chorioamnionotic membranes according to widely accepted, reproducible semi-quantitative scoring system [18]. Placental fetal and maternal responses were graded (grade 0 =  no inflammation, 1 =  early, 2 =  intermediate, 3 =  advanced) and staged (stage 0 =  no inflammation, 1 =  mild-moderate, 2 =  advanced), using these well-defined criteria [18]. Histological chorioamnionitis was defined as an abnormal maternal and/or fetal response. An abnormal fetal response was defined as grade/stage >0, whereas an abnormal maternal response was defined as grade/stage >1, as a grade 1 maternal response (indicative of focal or mild maternal inflammatory response) may not represent an established intra-amniotic infection.

Demographic and clinical data were collected at delivery. Maternal data included age, ethnicity, reported smoking, parity, pregnancy complications, premature rupture of membranes, length of labour, intrapartum fever, antibiotic exposure, antenatal steroids, and mode of delivery. Clinical chorioamnionitis was defined by the presence of at least two of the following features: intrapartum fever, uterine tenderness, maternal or fetal tachycardia, or malodorous amniotic fluid. Neonatal data included gestational age, sex, birth weight, head circumference, length, 1, 5 and 10 minute Apgar scores, ventilatory support, neonatal sepsis (defined as a single isolate cultured from a sterile site with suggestive clinical features and a treatment course of antibiotics [19]), time to discharge, and death.

### Sample Collection and Preparation

Immediately prior to delivery, maternal peripheral blood was collected into lithium-heparin, centrifuged for 10 mins at 3000 rpm and frozen for subsequent PCT and high sensitivity CRP (hsCRP) measurements. Placental specimens were examined by an experienced perinatal histopathologist, blinded to clinical and laboratory data. Tissue samples of HCA cases were cultured using standard media (chocolate agar and cysteine, lactose and electrolyte deficient plates) for aerobic bacteria, selective agar for mycoplasma and ureaplasma, and selective agar and enrichment broth for listeria.

Umbilical cord blood was collected in ethylene diamine tetra-acetic acid for full blood count and automated differential and lithium-heparin gel for plasma assays. Neonatal peripheral blood samples were collected when sepsis was clinically suspected by one or more suggestive clinical features: temperature instability, apnoea, bradycardia, or cardiovascular instability [19]. Neonatal blood samples were analysed for measurement of full blood count (0–24 h post-delivery) and CRP (first hour (0–1 h post-delivery), Day 1 (>1–24 h post-natal age), and Day 2 (24–48 h post-natal age).

### Inflammatory Mediator Assays

High sensitivity (hs)CRP assay was used to measure CRP in the maternal and cord blood samples as it was hypothesised that the effect of HCA on CRP would be modest and may not be detected using he conventional clinical assay, which has a lower limit of detection of 3–7 mg/L, depending on the assay used. High sensitivity CRP was measured by latex immunonephelometry (Dade Behring Nephelometer BN II, Marburg, Germany). The lower limit of detection was 0.15 mg/L, with values <0.15 mg/mL set to zero for statistical analysis. Procalcitonin was measured by the BRAHMS PCT sensitive immunoluminometric assay on a Berthold Technologies Lumat LB9507 luminometer (Bad Wildbad, Germany). The lower limit of detection was 0.01 ug/L and all samples had a value greater than this. The intra-assay co-efficient of variation was 4.8% and inter-assay co-efficient of variation was 5.6%. Cord and neonatal full blood counts were measured using a Beckman Coulter HmX analyzer (Fullerton, California, USA).

Postnatal CRP was measured only in neonates where clinically indicated up to the first 48 hours following delivery. For these samples, CRP was measured by enzymatic sandwich immunoassay using VITROS Chemistry Products CRP Slides (Ortho-Clinical Diagnostics, Rochester, USA). Until May 2005, the lower limit of detection for the assay was 7 mg/L. After this time, a more sensitive slide technique was used (lower limit of detection 3 mg/L). C-reactive protein values less than the lower limit of detection were analysed in two ways; with all values below the lower limit of detection set to 6.5 mg/L and 2.5 mg/L, respectively. The intra-assay co-efficient of variation was 3.1% and inter-assay co-efficient of variation was 2.5%. The maximum CRP value was the highest value for an infant within the first 48 h post-delivery, even if this was the only available CRP value.

### Statistical Analysis

Continuous data were summarised using non-parametric statistics: medians, interquartile ranges (IQR) and ranges (R), and Mann-Whitney U tests were used to compare groups. Categorical data were summarised using frequency distributions. Pearson’s chi-square tests or Fisher’s exact test were used to compare frequency distributions between groups.

Linear correlation and linear regression were used to explore the effect of HCA on maternal, cord and neonatal outcomes. Given the likely correlation between maternal and neonatal factors, analysis for maternal and neonatal outcomes used the number of infants delivered as the overall denominator. Regression models for maternal outcomes were adjusted for co-existing conditions associated with maternal inflammation; duration of PROM, intrapartum fever (>38°C), or duration of labour before delivery. Regression models for neonatal outcomes were adjusted for factors associated with elevated cytokine levels; gestational age, ‘small for gestational age’ (according to Australian normative data) [20], and duration of labour. In addition, cord and neonatal white cell count and absolute neutrophil count were adjusted for use of antenatal steroids [21]. Continuous outcomes, such as PCT and absolute neutrophil count, were log-transformed to achieve normality. Coefficient of determination (R^2^) was used to measure the proportion of variance explained by variables in the linear regression models. As there was no difference in parameters for multiple or singleton pregnancies, these were analysed together (data not shown). For all analyses, *P<*0.05 was considered statistically significant. Data was analysed using SPSS statistical software (version 15.0: Chicago, Illinois).

## Results

### Study Cohort

A total of 343 mothers, delivering 421 infants, satisfied inclusion criteria, of whom 247 mothers and their 325 infants had placental samples; analysis was therefore based on these deliveries. Two hundred and twenty two (68%) neonates were delivered preterm, 90 (28%) at 28–32 weeks gestational age, and 19 (6%) <28 weeks gestational age. The median gestational age was 34 weeks (range 24–42 weeks). Approximately two thirds were delivered by elective caesarean section (208, 64%), and one third (128, 39%) were multiple pregnancies.

### Placental Histology

Twenty-six placentae (of 247 examined, 10.5%) had HCA with a maternal response graded more than 1, and/or a fetal response [18]. The median gestational age of HCA cases was 30 weeks (IQR 27.5 to 32 weeks) and the majority (23, 88%) of HCA occurred in single gestation pregnancies. Placental culture results were available in 17 of 26 HCA cases, of which 11 (of 17, 65%) were positive. The most common isolates were *Mycoplasma hominis* (n = 5), *Ureaplasma urealyticum* (n = 2), and mixed bacterial species (n = 4) (Table 1).

**Table 1:** Description of cases with histologic chorioamnionitis (HCA).

Case	Maternalstage*	Maternal grade*	Fetal stage#	Fetal grade#	Maternal HCA	FetalHCA	GA(wks)	S/M	Placental sample	Bact.cult
**1**	1	0	2	1		1	34	M	partial	NA
**2**	1	1	2	2		1	25	S	full	*U. u*
**3**	1	1	1	1		1	33^+3^	M	full	neg
**4**	1	1	2	1		1	32	S	partial	MCB
**5**	1	1	1	1		1	40	S	partial	NA
**6**	1	1	1	1		1	39	S	partial	NA
**7**	2	1	1	1	1	1	25^+6^	S	partial	NA
**8**	2	1	0	0	1		32	S	full	*M. h*
**9**	2	1	1	1	1	1	31	S	full	neg
**10**	2	1	1	1	1	1	23	S	partial	MBF
**11**	2	1	1	1	1	1	40	S	partial	NA
**12**	2	2	2	1	1	1	30^+6^	S	partial	neg
**13**	2	2	0	0	1		27^+3^	S	full	*M. h*
**14**	2	2	1	1	1	1	29	S	partial	NA
**15**	2	2	3	2	1	1	28	M	partial	NA
**16**	3	2	1	1	1	1	28^+5^	S	partial	*M. h*
**17**	3	2	3	2	1	1	27	S	partial	neg
**18**	3	2	1	1	1	1	30	S	partial	NA
**19**	3	2	3	2	1	1	31	S	full	MCB
**20**	3	2	3	2	1	1	30	S	full	*C. a* *M. h*
**21**	3	2	3	2	1	1	24^+3^	S	full	neg
**22**	3	2	2	1	1	1	29	S	full	neg
**23**	3	2	2	1	1	1	28	S	full	NA
**24**	3	2	3	2	1	1	25	S	full	*M. h* *U. u*
**25**	3	2	3	2	1	1	31^+5^	S	full	MGF
**26**	3	4	1	1	1	1	35^+5^	S	partial	*S. a*
**Total**	**26**	**25**	**24**	**24**	**20**	**24**		**3M, 23S**		

Maternal and fetal inflammatory response were staged and graded according to criteria published by Redline *et al* [18]: *Maternal inflammatory response stage 0 = absent, 1 =  early, 2 =  intermediate, 3 =  advanced chorioamnionitis, grade 1 =  mild-moderate, 2 =  severe. #Fetal inflammatory response stage 0 =  absent, 1 =  early, 2 =  intermediate, 3 =  advanced funisitis, grade 1 =  mild-moderate, 2 =  severe. HCA was present if maternal response was grade/stage >1 and/or fetal response was grade/stage >0.

GA = gestational age, Single (S) = single gestation pregnancy, Multiple (M) = multiple gestation pregnancy, Bact. Cult. = bacterial culture result, *U. u* = *Ureaplasma* urealyticum, neg = negative, MCB = mixed coliform bacteria, *M. H* = *Mycoplasma* hominis, MBF = mixed bacterial flora, *C. A* = *Candida* albicans, MGF = mixed genital flora, *S. a* = *Staphylococcus* aureus, NA = not available.

### Maternal Characteristics

Univariate comparison of mothers with (n = 26) and without HCA (n = 299) showed that HCA was associated with Aboriginal or Torres Strait Island descent (6/26 (23%) versus 20/299 (7%); *P = *0.004), and maternal self-reported smoking (10/26 (39%) versus 42/299 (14%); *P = *0.001) (Table 2). Seven (27%) mothers with HCA had symptoms of clinical chorioamnionitis whereas 4 of 299 (1%) of those without HCA were symptomatic (*P<*0.001). Fifteen of 26 (58%) HCA cases had spontaneous vaginal deliveries, compared to 72/299 (25%) of cases without HCA (*P = *0.001). Antenatal steroids were more prevalent in mothers with HCA (15/26 (58%) versus 95/299 (32%); *P = *0.009.

**Table 2:** Demographic and clinical characteristics of maternal cases with and without histologic chorioamnionitis.

	*Histologic Chorioamnionitis*		
*Maternal characteristics*	No	Yes	*P value*
	n = 299 (92%)	n = 26 (8%)	
**Maternal age** * **(yrs)**	29	29	
	(26–34)	(22–34)	
	[14–45]	[16–43]	.779
**Maternal ethnic origin**			.010
Aboriginal/TSI	20 (7%)	6 (23%)	.004
Caucasian	241 (82%)	19 (73%)	
Other	33 (11%)	1 (4%)	
**Smoker**	42 (14%)	10 (39%)	.001
**Primigravid**	92 (31%)	7 (27%)	.393
**Antenatal steroids**	95 (32%)	15 (58%)	.009
**PROM** †	100 (34%)	19 (73%)	<.001
**Period of PROM (h)** † **#**	0.15	0.50	
	(0.05–0.24)	(0.09–3.45)	
	[0–13]	[0–9]	.014
**Antibiotics for PROM**	31 (11%)	11 (42%)	<.001
**Antibiotics prior labour onset**	26 (9%)	11 (42%)	<.001
**Antibiotics during labour**	50 (17%)	13 (50%)	<.001
**Clinical chorioamnionitis**	4 (1%)	7 (27%)	<.001
**Intrapartum fever >38°C**	4 (1%)	6 (23%)	<.001
**Mode of delivery**			.001
Spontaneous VD	72 (25%)	15 (58%)	
Assisted VD	19 (7%)	3 (12%)	
Emergency CS	3 (1%)	–	
Elective CS	200 (68%)	8 (31%)	
**Any labour** *(1^st^ +2^nd^ stage)*	133 (45%)	23 (89%)	<.001
**Length of labour (h)** * **##**	5.73	5.90	.586
	(3.36–9.70)	(3.37–11.80)	
	[0.02–25.58]	[1.93–30.17]	
**Multiple births**			
Multiples	125 (43%)	3 (11%)	.002

Frequencies expressed as n(%) unless specified.

* Median (Interquartile Range) [Range].

† PROM = Premature rupture of membranes.

#PROM cases only ## Labour cases only.

### Neonatal Characteristics

Newborns born after HCA-affected pregnancies were more likely to be preterm (23/26 (89%) versus 199/299 (68%); *P = *0.028) and had a lower gestational age (median 30 (range 23–40) weeks versus 34 (26–42) weeks, respectively, *P<*0.001). More HCA cases were born at 28–32 weeks GA (7/26 (27%) versus 12/299 (4%); *P<*0.001), and <28 week GA (11/26 (42%) versus 79/299 (27%); *P<*0.001) (Table 3). The only case with proven early-onset sepsis (EOS) was excluded from analysis as infection elevates CRP and PCT, potentially confounding the effects of HCA on inflammatory markers, which was the primary research question.

**Table 3:** Demographic and clinical characteristics of neonates with and without histologic chorioamnionitis.

	***Histologic Chorioamnionitis***		
***Neonatal characteristics***	No	Yes	***P value***
	n = 299 (92%)	n = 26 (8%)	
**Gestational age** *	34	30	
	(31–38)	(27–32)	
	[26–42]	[23–40]	<.001
<28w	12 (4%)	7 (27%)	<.001
28–32w	79 (27%)	11 (42%)	
32–37w	108 (37%)	5 (19%)	
37+w	95 (32%)	3 (12%)	
**Preterm**	199 (68%)	23 (89%)	.028
**Male gender**	132 (45%)	10 (62%)	.527
Birth weight*	2158	1575	
	(1510–3081)	(1048–2151)	
	[495–4945]	[585–3565]	.002
Expected birthweight*	2282	1403	
	(1581–3187)	(999–1432)	
	[865–3739]	[591–3463]	<.001
Head circumference*	31	29	
	(28–34)	(25–30)	
	[21–39]	[21–37]	.001
**Length** *	45	40	
	(40–49)	(36–45)	
	[30–59]	[30–51]	.001
**SGA** *(<10%ile)* ‡	41 (14%)	2 (8%)	.551
**Apgar Scores**			
Apgar 1 minute <6	53 (18%)	12 (46%)	.001
Apgar 5 minute <6	10 (3%)	3 (12%)	.029
**Any ventilation**	133 (45%)	19 (73%)	.005
**Neonatal sepsis** *(early & late onset)*	17 (6%)	5 (19%)	.009
**Neonatal early onset sepsis** *(<72* *h)*	1 (<1%)	-	.768
**Time to discharge** (d)*	19	32	
	(5–39)	(14–62)	
	[0–123]	[0–97]	.014
**Neonatal death**	1 (<1%)	2 (8%)	<.001

Frequencies expressed as n(%) unless specified *Median (Interquartile Range) [Range].

‡ SGA = Small for gestational age (<10^th^ percentile).

### Effect of HCA on Maternal hsCRP and Procalcitonin

Maternal hsCRP level was significantly higher in mothers with HCA (median 26 mg/L (IQR 12.6–61.3, range 2.0–107) versus 5.6 (IQR 2.4–13.6; range 0–108) mg/L; *P<*0.001), but there was no difference in median PCT (0.046, R 0.014–0.342 versus 0.037, R 0.011–1.565, *P* = NS). Multivariable linear regression, adjusting for duration of prolonged rupture of membranes, length of labour and intrapartum fever showed a significant association between HCA and maternal hsCRP (*P<*0.001), and maternal hsCRP was significantly higher in mothers with HCA compared to those without HCA (OR 2.86; 95% CI 1.47–5.57; *P = *0.002). There was no statistically significant relationship between HCA and maternal PCT.

### Effect of HCA on Umbilical Cord hsCRP and Procalcitonin

The median cord PCT level was significantly higher in subjects with HCA (0.293 (IQR 0.08–0.44, R 0.05–27.37) ug/L versus 0.064 (IQR 0.05–0.10, range 0.01–5.24) ug/L; *P<*0.001), as was hsCRP (0, 0–2.9; 0–63.9) mg/L versus (0, 0–0; 0–45.6, *P*<0.001). For cord PCT, multivariable linear regression showed gestational age, being small for gestational age, and length of labour accounted for 30.7% of PCT variation, with HCA contributing a further 10.5% variance; HCA was a significant variable in the model (*P = *0.001). For cord hsCRP, gestational age, small for gestational age and length of labour accounted for 3.7% of the increase in cord hsCRP and HCA contributed a further 9% variance; HCA was a significant variable in the model (*P<*0.001).

### Effect of HCA on Cord and Neonatal Haematological Parameters

No significant differences were observed between cord haematological parameters in subjects with and without HCA (Table S1). Neonatal peripheral blood absolute neutrophil count was similar in HCA (2.4 (range (1.0–3.3)×10^9^/L) than in those without HCA (2.0 (range 0.9–4.2)×10^9^/L, *P = NS*). Gestational age, small for gestational age, length of labour, and antenatal steroid exposure affected white blood count and absolute neutrophil count, and the relationship between HCA and neonatal white blood count and absolute neutrophil count was further examined using multivariable linear regression. For neonatal white cell count, these factors accounted for 8.6% of variation, with HCA contributing a further 7.1%; HCA was significant in the regression model (*P<*0.001). For neonatal absolute neutrophil count, the same variables accounted for 23.8% of variation; HCA contributed a further 3.4% and remained significant (*P = *0.002).

### Histological Chorioamnionitis and Neonatal CRP

C-reactive protein was measured in the peripheral blood of 217 neonates (Table S2). Twenty-three of 26 (89%) of neonates exposed to HCA and 191/299 (64%) of unexposed neonates had CRP measured within the first 48 h of life. Given the proportion of missing data for CRP, particularly in the non-HCA group, regression analysis was only performed for maximal CRP for each infant recorded within the first 48 h after delivery.

**Figure 1:**
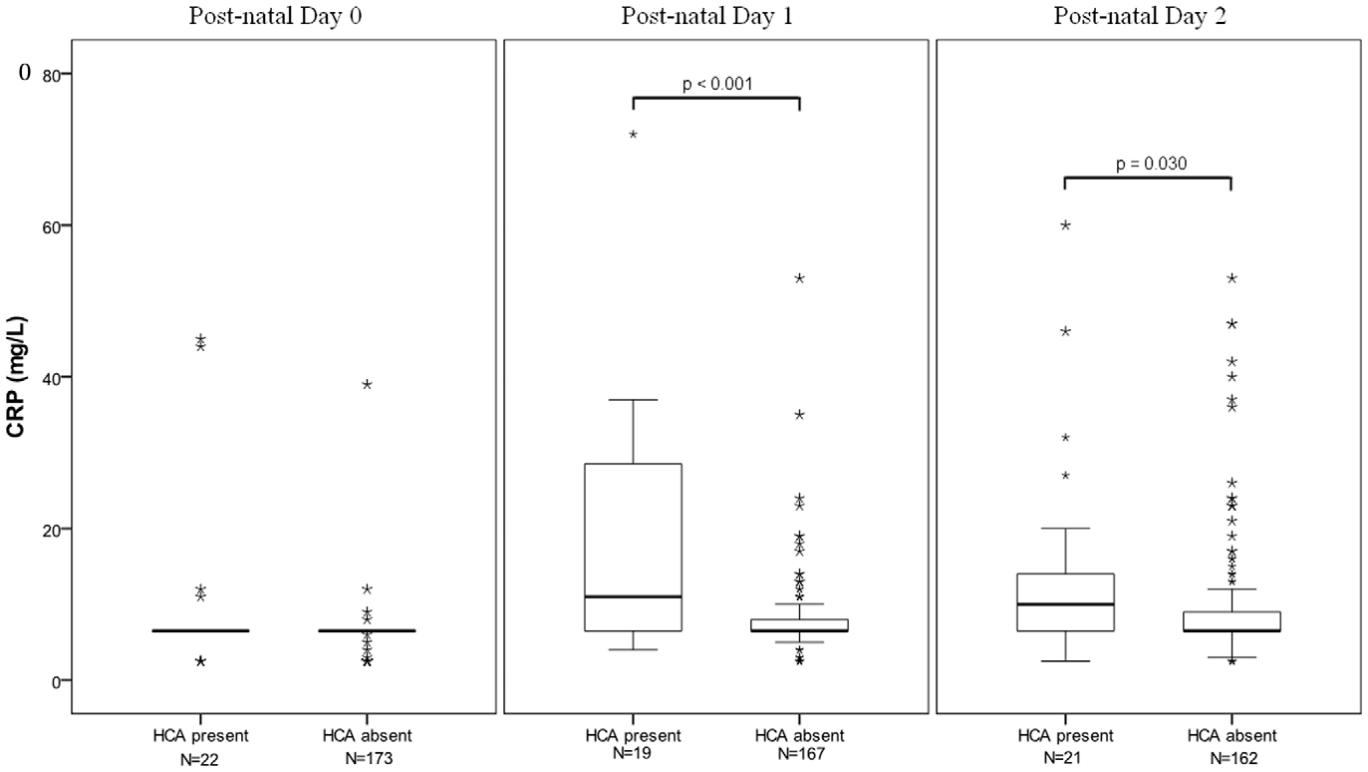
C-reactive protein (CRP) concentrations in newborns with and without histologic chorioamnionitis (HCA) in the first hour (Day 0), 1–24 hours (Day 1) and 24–48 hours (Day 2) following delivery.

Figure 1 shows neonatal CRP results during the first 48 h of life. Linear regression for the effect of HCA on neonatal maximal CRP in the first 48 h showed gestational age, small for gestational age and length of labour accounted for only 1.0% of variation in postnatal CRP. The addition of HCA accounted for a further 9.0%; the contribution of HCA to CRP was significant (*P<*0.001). Analysis of the timing of maximal postnatal CRP in neonates exposed to HCA showed a significant relationship between HCA and maximal CRP at Day 1 (*P<*0.001) and Day 2 (*P = *0.03), but no significant relationship with maximal CRP levels at birth.

## Discussion

These findings highlight the possible relationship between HCA and inflammatory and haematological markers in the maternal, cord and neonatal circulation. In the majority of mothers, HCA was asymptomatic and associated with a modest increase in hsCRP, but not in PCT levels. In umbilical cord blood, HCA was associated with increased PCT and hsCRP, but did not affect cord white cell count or absolute neutrophil count. However, there was a significant increase in inflammatory and haematological markers in infants exposed to HCA without early onset sepsis, peaking at 24 h of age. Histological choriomanionitis may therefore result in sustained inflammatory changes in the neonatal circulation, in the absence of microbiologically diagnosed infection.

The observation of higher CRP but no difference in PCT in the mothers with HCA is in keeping with many previous studies, although data are not uniformly consistent and most studies are on pregnancies complicated by prolonged rupture of membranes [22], [23]. The methodology of studies investigating maternal CRP in diagnosing HCA varies, particularly in relation to the CRP cut-off values [16], and there are few data on hsCRP [22]. Although there appears to be significant differences in maternal CRP levels in those with and without HCA, the considerable overlap between these groups may limit the clinical utility of CRP in diagnosing HCA [23]. In infants exposed to HCA we found significant differences in PCT, and to a lesser extent, hsCRP concentrations cord blood. The discrepant CRP and PCT findings between mothers and infants may reflect differential regulation of CRP and PCT production by placental cytokines [24], [25]. In addition the kinetics of CRP and PCT differ; PCT is synthesised more quickly and CRP degrades more slowly [26]. Serial CRP and PCT measurements postnatally in infants exposed to HCA may be informative.

Perinatal inflammation is associated with increased umbilical cord inflammatory cytokines including interleukin (IL)-6 and acute phase reactants such as CRP [26], [27], [28], but the impact on inflammatory markers in the early neonatal period is less well-described. Placental inflammation in extremely preterm newborns is associated with elevation in concentrations of acute phase reactants during the first three days after birth [29]. Our data suggest that even in infants without obvious evidence of bacterial infection, the response to perinatal inflammation persists postnatally and may be sufficient to cause clinically significant changes in acute phase reactants. The peak CRP occurred on day 1, reflecting the kinetics of CRP production - detectable within 6 h and peaking at approximately 48 h [30].

Procalcitonin, like CRP, is regulated by cytokines that increase early in HCA [31]. In adult intensive care, PCT may have advantages over CRP in differentiating bacterial infection from other causes of inflammation [32]. Here we report that cord PCT concentrations were significantly higher in neonates exposed to HCA. Although our study aimed to investigate the more common influences on PCT and neonatal PCT is not routinely measured, our data suggest that gestational age, small for gestational age and length of labour and HCA account for almost half of the variation in cord PCT levels, which therefore may limit its utility in the diagnosis of early neonatal infection. There are few data on PCT and HCA. One study reported that HCA did not influence cord PCT levels, but was associated with a post-natal increase in PCT at 72 h and 7 days [15]. Although higher PCT levels are reported in infants who subsequently develop sepsis [26], these studies did not examine placental histology.

Histological chorioamnionitis was associated with increased absolute neutrophil in the first 48 h of life, consistent with other data [33]. There was marked overlap in WCC and ANC between HCA and non-HCA groups, limiting their clinical utility in identifying HCA and in diagnosing EOS in HCA-exposed infants.

The strengths of our study include quantification of HCA by an experienced perinatal histopathologist, blinded to outcomes and analysis of parameters commonly used in clinical practice in maternal, fetal and post-natal samples. We acknowledge some limitations, most importantly the non-representative nature of our sample, which reflected the resources available to recruit mothers and infants and to process samples. We therefore enrolled a relative over-representation of moderately preterm infants and of those born by caesarean section. Therefore, and despite the overall sample size, there were fewer cases of HCA than we have previously observed in a more preterm cohort drawn from the same population [34], and thus there was some reduced power and limitation in the number of variables used in regression modelling. The lower frequency of HCA was likely due in part to the over-representation of elective caesarean sections and elective deliveries for multiple pregnancies, and under-representation of spontaneous vaginal deliveries and extremely preterm infants. As HCA predominantly occurred as expected in spontaneous preterm, singleton vaginal deliveries, the findings reflect the overall maternal and neonatal response to HCA, but inclusion of extremely preterm infants would be additionally informative, as this group has a higher incidence of HCA and of neonatal infection. Further studies in a larger unselected preterm population are therefore necessary to confirm our findings. Placental samples were not available from all deliveries, although this is less likely to introduce a systematic bias, as placental histology is part of routine clinical practice in all deliveries less than 34 weeks gestational age and from all placentae successfully collected as part of the study, irrespective of gestational age or clinical features. The higher prevalence of antenatal steroids in the mothers with HCA may have diminished the extent of inflammation, thus dampening the effect of HCA on inflammatory biomarkers. Despite this, our study did find higher CRP concentrations in newborns exposed to HCA. Maternal blood contamination of cord blood samples is also unlikely to have been a major limitation; our sampling protocols aim to reduce maternal blood contamination of cord blood, which largely reflects the fetal circulation at late gestation [35]. As postnatal CRP was only measured on infants who were clinically suspected of having sepsis, a significant proportion of neonates did not have CRP measured postnatally, precluding regression analysis of post-natal CRP data. It would be of interest for future studies to investigate inflammatory markers in all infants exposed to HCA, irrespective of whether infection was clinically suspected.

### Conclusions

Histological chorioamnionitis has differential effects on maternal and cord/neonatal inflammatory markers. In the newborn exposed to HCA, CRP, white cell count and absolute neutrophil count are increased when compared to newborns without HCA. This may confound the interpretation of these common diagnostic markers of early neonatal sepsis. Further investigations in larger unselected populations of preterm infants, with serial measurement for a longer period of hsCRP and PCT, irrespective of suspected sepsis, would be informative about the effects of HCA on postnatal inflammatory markers and would refine the use of plasma and haematological markers in the diagnosis of neonatal infection.

## Supporting Information

Table S1 **Cord and neonatal haematological parameters in subjects with and without histological chorioamnionitis.**
(DOCX)

Table S2 **Availability of neonatal CRP measures within the first 48 hours of delivery.**
(DOCX)
