# Supplementary material for: Inflammatory and Haematological Markers in the Maternal, Umbilical Cord and Infant Circulation in Histological Chorioamnionitis
Source: PLoS One. 2012 Dec 13;7(12):e51836. doi: 10.1371/journal.pone.0051836 (PMC3521712; doi:10.1371/journal.pone.0051836)
Supplement: Table S1 — Cord and neonatal haematological parameters in subjects with and without histological chorioamnionitis. (DOCX) [file pone.0051836.s001.docx]

**Table S1 Cord and neonatal haematological parameters in subjects with and without HCA**

|  |  | ***Histologic Chorioamnionitis**** | | |  |  |
| --- | --- | --- | --- | --- | --- | --- |
|  |  | No |  | Yes |  | ***P-value*** |
|  |  | n=299 (92%) |  | n=26 (8%) |  |  |
| ***Cord blood:*** |  |  |  |  |  |  |
| White cell count |  | 8.41 |  | 8.16 |  |  |
|  |  | (6.24-10.46) |  | (4.64-9.73) |  |  |
|  |  | [1.7-21.0] |  | [3.0-21.5] |  | .357 |
| Platelet count |  | 262 |  | 236 |  |  |
|  |  | (220-322) |  | (196-294) |  |  |
|  |  | [5-549] |  | [106-338] |  | .201 |
| Neutrophil count |  | 2.05 |  | 2.36 |  |  |
|  |  | (0.93-4.21) |  | (1.04-3.29) |  |  |
|  |  | [0.2-13.1] |  | [0-10.6] |  | .840 |
| Nucleated red blood cell count |  | 9 |  | 9 |  |  |
|  |  | (4-23) |  | (5.5-19.5) |  |  |
|  |  | [0-312] |  | [3-68] |  | .839 |
| ***Neonatal blood*** |  |  |  |  |  |  |
| White cell count |  | 9.2 |  | 10.3 |  |  |
|  |  | (7.1-12.5) |  | (6.2-18.0) |  |  |
|  |  | [2.1-24.5] |  | [2.9-44.8] |  | .478 |
| Platelet count |  | 248 |  | 246 |  |  |
|  |  | (200-281) |  | (198-310) |  |  |
|  |  | [27-500] |  | [83-580] |  | .751 |
| Neutrophil count |  | 3 |  | 4.5 |  |  |
|  |  | (1.7-5.7) |  | (2.1-8.7) |  |  |
|  |  | [0.1-17.8] |  | [0.1-26.4] |  | .054 |
| Nucleated red blood cell count |  | 9 |  | 17 |  |  |
|  |  | (4-22) |  | (3-33) |  |  |
|  |  | [0-416] |  | [0-92] |  | .326 |
