# Supplementary material for: Inflammatory and Haematological Markers in the Maternal, Umbilical Cord and Infant Circulation in Histological Chorioamnionitis
Source: PLoS One. 2012 Dec 13;7(12):e51836. doi: 10.1371/journal.pone.0051836 (PMC3521712; doi:10.1371/journal.pone.0051836)
Supplement: Table S2 — Availability of neonatal CRP measures within the first 48 hours of delivery. (DOCX) [file pone.0051836.s002.docx]

**Table S2 Availability of neonatal CRP measures within the first 48 hours of delivery**

|  |  | ***Histologic Chorioamnionitis*** | | |  |
| --- | --- | --- | --- | --- | --- |
| ***Availability of CRP measures*** |  | No |  | Yes |  |
|  |  | n=299 |  | n=26 |  |
|  |  |  |  |  |  |
| Any CRP measures available: |  | 191 (64%) |  | 23 (89%) |  |
| Maximal CRP available: |  | 191 (64%) |  | 23 (89%) |  |
|  |  |  |  |  |  |
| CRP measures available: |  |  |  |  |  |
| First hour (birth-1 hr) |  | 173 (58%) |  | 22 (85%) |  |
| Day 1 (1 - 24hrs) |  | 167 (56%) |  | 19 (73%) |  |
| Day 2 (24 - 48hrs) |  | 162 (54%) |  | 21 (81%) |  |
|  |  |  |  |  |  |
| CRP measures less than 7mg/L: |  |  |  |  |  |
| First hour |  | 151 (87%)* |  | 14 (64%)* |  |
| Day 1 |  | 92 (55%)* |  | 7 (37%)* |  |
| Day 2 |  | 84 (52%)* |  | 6 (29%)* |  |
|  |  |  |  |  |  |
| CRP measures less than 3mg/L: |  |  |  |  |  |
| First hour |  | 24 (14%)* |  | 4 (18%)* |  |
| Day 1 |  | 15 (9%)* |  | 0 (0%)* |  |
| Day 2 |  | 9 (6%)* |  | 1 (5%)* |  |
|  |  |  |  |  |  |

% of total CRP measures available at that time point (i.e. First hour, Day 1 or Day 2)
